# Bounded Cost Path Planning for Underwater Vehicles Assisted by a Time-Invariant Partitioned Flow Field Model

**DOI:** 10.3389/frobt.2021.575267

**Published:** 2021-07-14

**Authors:** Mengxue Hou, Sungjin Cho, Haomin Zhou, Catherine R. Edwards, Fumin Zhang

**Affiliations:** ^1^ Electrical and Computer Engineering, Georgia Institute of Technology, Atlanta, GA, United States; ^2^ Department of Guidance and Control, Agency for Defense Development, Daejeon, South Korea; ^3^ School of Mathematics, Georgia Institute of Technology, Atlanta, GA, United States; ^4^ Skidaway Institute of Oceanography, University of Georgia, Savannah, GA, United States

**Keywords:** robotic path planning, graph search method, bounded cost search, parameter identification, underwater vehicle

## Abstract

A bounded cost path planning method is developed for underwater vehicles assisted by a data-driven flow modeling method. The modeled flow field is partitioned as a set of cells of piece-wise constant flow speed. A flow partition algorithm and a parameter estimation algorithm are proposed to learn the flow field structure and parameters with justified convergence. A bounded cost path planning algorithm is developed taking advantage of the partitioned flow model. An extended potential search method is proposed to determine the sequence of partitions that the optimal path crosses. The optimal path within each partition is then determined by solving a constrained optimization problem. Theoretical justification is provided for the proposed extended potential search method generating the optimal solution. The path planned has the highest probability to satisfy the bounded cost constraint. The performance of the algorithms is demonstrated with experimental and simulation results, which show that the proposed method is more computationally efficient than some of the existing methods.

## 1 Introduction

Over the last few decades, autonomous underwater vehicles (AUVs) have been employed for ocean sampling (Leonard et al., 2010; Smith et al., 2010), surveillance and inspection (Ozog et al., 2016; Xiang et al., 2010), and many other applications. Ocean flow is the dominant factor that affects the motion of AUVs (Zhang et al., 2016). Ocean flow dynamics vary in both space and time, and can be represented as geophysical Partial Differential Equations (PDEs) in ocean circulation models (e.g., the Regional Ocean Modeling System (ROMS, Shchepetkin and McWilliams, 2005; Haidvogel et al., 2008) and the Hybrid Coordinate Ocean Model (HYCOM, Chassignet et al., 2007)). While these models can provide flow information over a large spatial domain and forecast over several days, the available flow field forecast may still contain high uncertainty and error. The uncertainty comes from multiple sources, including the incomplete physics or boundary conditions (Haza et al., 2007; Griffa et al., 2004) and even terms in the equations themselves (Lermusiaux, 2006). In addition, the high complexity of the flow dynamics makes solving these PDEs computationally expensive. Data-driven flow models (Mokhasi et al., 2009; Chang et al., 2014) can provide short-term flow prediction in a relatively smaller area with significantly lower computational cost, and can be more suitable for supporting real-time AUV navigation, particularly for systems with strong gradients and/or high uncertainty.

Path planning is one of the crucial and fundamental functions to achieve autonomy. Two key considerations for an AUV path planner are computational efficiency and path quality. The path planning strategy should be computationally efficient so that the time for generating a path can be kept to a minimum. When these methods are sufficiently fast, path planning can be performed in near-real time, generating a feasible solution in minutes while the AUV has surfaced to get a GPS fix and communicate with shore. Advantages of real time path planning are that more recent information, including the real time data from the vehicle, can be incorporated in path planning. Hence there will be less planning error due to outdated information (Leonard et al., 2010). At the same time, it is desired that the path planning algorithm has theoretical guarantee on the quality of the generated path.

Most path planning algorithms aim to design optimal path minimizing certain cost, for example, those associated with engineering or flight characteristics (battery life, travel time) or scientific value (e.g., distance relative to other assets or spacing of relevant processes). Algorithms that have been applied to AUV optimal path planning include: 1) graph-based methods such as the A* method (Rhoads et al., 2012; Pereira et al., 2013; Kularatne et al., 2017, 2018) and the Sliding Wavefront Expansion (SWE) (Soulignac, 2011); 2) sampling-based methods like the Rapidly exploring Random Trees (RRTs) (Kuffner and LaValle, 2000; Cui et al., 2015), RRT* (Karaman and Frazzoli, 2011) and informed RRT* (Gammell et al., 2018); 3) methods that approximate the solution of HJ (Hamilton-Jacobi) equations, such as the Level Set Method (LSM) (Subramani and Lermusiaux, 2016; Lolla et al., 2014), and 4) the evolutionary algorithms, including the particle swarm optimization methods (Roberge et al., 2012; Zeng et al., 2014), and the differential evolution methods (Zamuda and Sosa, 2014; Zamuda et al., 2016). See (Zamuda and Sosa, 2019; Zeng et al., 2015; Panda et al., 2020) for a comprehensive review on the existing AUV path planning methods. However, the computational cost of the above mentioned methods could be high, especially in cases where the AUV deployment domain is large.

Using a regular grid to discretize the flow field can result in unnecessary large number of cells, which increases the computational burden of the graph search methods. Since the flow speed in adjacent cells is usually similar, we partition the flow field into piece-wise constant subfields, within each the flow speed is a constant vector, and introduce the Method of Evolving Junctions (MEJ) (Zhai et al., 2020) to solve the optimal path planning problem. MEJ solves for the optimal path by recasting the infinite dimensional path planning problem into a finite dimensional optimization problem through introducing a number of junction points, defined as the intersection between the path and the region boundaries. Hence the computation cost of MEJ is significantly lower than other optimal path planning methods, especially when the flow field is partitioned into a small number of cells (Zhai et al., 2020). We identify Soulignac. (2011) as the work closest related to ours, in which sliders, defined as points sliding on the partitioned region boundaries, are introduced to describe the wavefront expansion of the graph search methods. In each iteration of the wavefront expansion, each slider’s position on the wavefront is derived by minimizing the travel cost in a single cell, and then the planned path is computed by the backtracking of wavefronts. Both MEJ and SWE are based on a novel parameterization of the path by introducing the junctions and the sliders, that were discovered independently by the two research groups. The main difference between MEJ and SWE lies in that MEJ solves for junction positions by formulating a non-convex optimization problem, and derives the global minimizer by intermittent diffusion (Li et al., 2017), which intermittently adds white noise to the gradient flow, while SWE solves for slider positions by graph search methods. MEJ has been justified to find the global minimizer with probability 1. However, since the method does not pose any structure in the search, the computational cost of MEJ could be less favorable compared to SWE if the number of cells scales up.

To reduce the computational cost of the path planning problem, the search can be reduced to find paths with total cost less than an upper bound. Stern et al. (2014) present two algorithms to solve the bounded cost search problem: the Potential Search (PTS) and the Anytime Potential Search (APTS). The PTS method defines the Potential Ordering Function, which is an implicit evaluation of the probability that a node is on a path satisfying the bounded cost constraint, and iteratively expands the nodes in the graph with the highest Potential Ordering Function value. The wavefront expansion terminates when the goal node has been expanded, and the path is found by backtracking of the wavefronts. The APTS method runs the PTS algorithm iteratively to improve on the incumbent solution, with the upper bound on total cost lowered in each iteration of the algorithm. Later work (Thayer et al., 2012) improves on the PTS method by minimizing both the potential and an estimation of the remaining search effort, so that the bounded cost search problem will be solved faster.

In this paper, our first objective is to develop a data-driven computational flow model that approximates the true flow field in the region of interest to assist AUV path planning. The proposed data-driven flow model divides the flow field into cells, within which the flow is represented as a single flow vector. The optimal flow cell partition and initial values of the flow vectors in each cell are derived from prior flow information, from numerical ocean models or from observations. To improve model accuracy, AUV observational data can be incorporated into the data-driven model in near-real time, for example, in the form of observed or estimated velocities (Chang et al., 2015). Here, we design a learning algorithm that estimates the flow field parameters based on the AUV path data. Our second objective is to develop an algorithm that solves the AUV bounded cost path planning problem. Given that the vehicle is traveling in a flow field represented by the data-driven computational model, the goal is to design a path that connects AUV initial position with goal position with the highest probability to have travel cost less than a pre-assigned upper bound. By introducing the key function, which is an implicit evaluation function of the probability that a path satisfies the bounded cost constraint, the optimal path is computed by searching for the nodes with lowest key function value using an informed graph search method.

The main novelty of this work is introducing the modified PTS method to solve the bounded cost search problem. Unlike the PTS method (Stern et al., 2014), which assumes that the branch cost of the graph is known exactly, our method deals with problems where the branch cost of the graph is uncertain. Given assumptions on the distribution of cost-of-arrival and cost-to-go, we prove that the proposed algorithm guarantees optimality of the planned path, that is, the planned path has the highest probability of satisfying the bounded cost constraint. To the best of our knowledge, this is the first time that the optimality of the modified PTS solution to bounded cost problems is proved. The proposed bounded cost path planning method can be viewed as an extension to MEJ. Compared to MEJ, the modified PTS algorithm is computationally more efficient, since a graph search method is adopted to search for the junction positions. At the same time, optimality of the planned path can be theoretically justified. The major benefit of the proposed bounded cost path planning algorithm lies in that it plans a path faster, while at the same time still guarantees the path quality. This paper is a significant extension of the conference proceeding (Hou et al., 2019), which proposes a flow partition method that approximates the flow field by a set of cells of uniform flow speed. The main extensions of this paper are that taking advantage of the flow model proposed in (Hou et al., 2019), we propose the modified PTS method to solve the AUV bounded cost path planning problem, and present theoretical justification on the optimality of the proposed PTS method. The proposed bounded cost search method is potentially applicable for all bounded cost path planning problems with uncertain branch cost.

We believe the proposed data-driven flow modeling and bounded cost path planning methods are well-suited for path planning of underwater glider deployment near Cape Hatteras, NC, a highly dynamic region characterized by confluent western boundary currents and convergence in the adjacent shelf and slope waters. While deployed, the gliders are subject to rich and complex current fields driven by a combination and interaction of Gulf Stream, wind, and buoyancy forcing, with significant cross-shelf exchange on small spatial and temporal scales (Savidge et al., 2013a, Savidge et al., 2013b) that would be highly difficult to sample using traditional methods. Path planning must consider spatial variability of the flow field. Because spatial gradients are significant, real-time path planning is critical to take advantage of real-time data streams. Through simulated experiments, we demonstrate the performance of applying the proposed algorithms to underwater glider deployment in this area, and show that the proposed algorithm is more computationally efficient than A* and LSM.

## 2 Problem Formulation

### 2.1 Vehicle Dynamics

Let $F_{R}:D\to {\mathbb{R}}^{2}$ represent a spatially distributed vector field for the ambient flow velocity, where $D\in {\mathbb{R}}^{2}$ is the domain of interest. Let $\left[T_{0},T_{f}\right]$ be the AUV deployment time interval. The AUV model is described as

$$\dot{x}=F_{R}\left(x\right)+V_{R}\Psi_{C}\left(t\right),$$ (1)

where x∈D denotes vehicle position. $V_{R}$ is the through-water speed of the vehicle, and $\Psi_{C}\left(t\right)={\left[\cos\psi_{C},\sin\psi_{C}\right]}^{T}$ is a unit vector that represents the direction of the vehicle motion along heading angle $\psi_{\text{C}}$.

Assumption 2.1 : During the operation, $V_{R}$ is an unknown constant.

Remark 2.1 : Actual vehicle speed may depend on a number of factors that affect an AUV’s speed, including water depth, efficiency of propulsion, and bio-fouling. These effects are difficult to estimate. Hence the vehicle forward speed is assumed to be an unknown constant.

Assumption 2.2 : We assume that the heading $\Psi_{C}\left(t\right)$ can be controlled for all time t, and the vehicle trajectory x(t) can be measured or estimated for all time.

Remark 2.2 : Though the actual location of a vehicle may only be known occasionally when the vehicle is underwater, the trajectory of the vehicle can be estimated through localization algorithms, which incorporates the known locations and the heading angle commands as inputs to generate the optimal state estimation.

Assumption 2.3 : We assume the flow field is time-invariant throughout the deployment.

Remark 2.3 : Even though there are existing work that considers the time-variant flow field in solving the AUV planning problem, such as (Eichhorn, 2013; Lolla et al., 2014; Zamuda and Sosa, 2014), we make this assumption due to the patterns of the flow field in this domain. In the domain of interest considered in this paper, which is near Cape Hatteras, NC, the current field is driven by a combination and interaction of Gulf Stream, wind, and buoyancy forcing (Savidge et al., 2013a, Savidge et al., 2013b). Because magnitude and spatial gradients of the flow field are significant relative to the temporal variation of the flow field (mostly the tidal flow component), time variation of the flow does not have a significant influence over the planned path.

### 2.2 Data-Driven Flow Modeling

Flow speed at neighboring grid points often exhibits similarity in both strength and direction. Hence we assume that at the time scale of an AUV deployment, the flow field can be divided into finite number of regions ${\left\{{ℛ}_{i}\right\}}_{i\in I_{R}}$, with the union of all cells being the domain, $\underset{i\in I_{R}}{\cup }\left\{{ℛ}_{i}\right\}=D$. The regions are separated by continuous boundary curves. boundary curves ${\left\{f_{i,j}\right\}}_{i,j\in I_{R}}$, and $f_{ij}\left(x\right)=0$ is the one dimensional compact boundary of the region ${ℛ}_{i}$ and ${ℛ}_{j}$. We define an indicator function $\phi^{i}\left(x\right)$ as follows:

$$\phi^{i}\left(x\right)=1\left\{x\in {ℛ}_{i}\right\}=\{\begin{array}{cc} 1 & \text{if}\;x\in {ℛ}_{i}\\ 0 & \text{otherwise.} \end{array}$$

This function indicates whether x is in ${ℛ}_{\alpha }$. Let $\phi :{\mathbb{R}}^{2}\to {\mathbb{R}}^{N}$ be defined as $\phi \left(x\right)={\left[\begin{array}{ccc} \phi^{1}\left(x\right) & \ldots & \phi^{N}\left(x\right) \end{array}\right]}^{T}$. Then ϕ(x) are a set of spatial basis functions of D.

In order to compute the partition, which is represented by the basis functions ϕ. We need to use prior information of the environment obtained either from forecast data of the existing ocean models, or from historical datasets. Let $F_{0}:D\times \left[T_{0},T_{f}\right]\to {\mathbb{R}}^{2}$ denote the discretized flow map forecast available on a set of grid points in D, and let $y\in {\mathbb{R}}^{4},y={\left[x^{T},F_{0}{\left(x,t\right)}^{T}\right]}^{T}$ denote the vector at position x and time *t*. We can define a distance function as $\text{dist}:{\mathbb{R}}^{4}\times {\mathbb{R}}^{4}\to \mathbb{R}$ as ${\text{dist}}^{2}\left(y,y^{\prime }\right)={\left(y-y^{\prime }\right)}^{T}Q\left(y-y^{\prime }\right),$ where $y,y\prime \in {\mathbb{R}}^{4}$, Q is a weight matrix. For each cell ${ℛ}_{i}$, let ${\bar{r}}_{i}$ represents its center, and let $\nu_{i}$ be the flow vector in this cell. Our goal is to find the optimal values of ϕ(x) and ${\left\{\nu_{i}\right\}}_{i\in I_{R}}$ by solving the following optimization problem:

$$\underset{\phi \left(x\right),{\left\{\nu_{i}\right\}}_{i\in I_{R}}}{\min}J=\underset{i\in I_{R}}{\sum }\underset{x\in {ℛ}_{i}}{\sum }\underset{t\in \left[T_{0},T_{f}\right]}{\sum }{\text{dist}}^{2}\left(y\left(x,t\right),{\left[{\bar{r}}_{i}^{T},\nu_{i}^{T}\right]}^{T}\right).$$ (2)

After the optimal partition is computed from forecast or historical datasets, we need to compute the strength of the flow in each partition based on the path information of the AUV while moving through the flow field. Again, we assume that the true flow field is constant in each of the partitioned cell. Let $\theta \in {\mathbb{R}}^{2N}$ be the true flow vectors in all of the partitioned cells, $\theta =\left[\begin{array}{c} \theta_{1}\\ \theta_{2} \end{array}\right]=\left[\begin{array}{ccc} \theta_{1}^{1} & \ldots & \theta_{1}^{N}\\ \theta_{2}^{1} & \ldots & \theta_{2}^{N} \end{array}\right]$, with $\theta^{i}={\left[\theta_{1}^{i},\theta_{2}^{i}\right]}^{T}$ denoting the flow vector in partitioned region ${ℛ}_{\alpha }$. Then the partitioned flow field can be represented as

$$F_{R}\left(x\right)=\theta \phi \left(x\right).$$ (3)

To estimate the true flow field $F_{R}$, we use the AUV path data x(t). Let

$$\xi \left(t\right)=\left[\begin{array}{c} \xi_{1}\left(t\right)\\ \xi_{2}\left(t\right) \end{array}\right]=\left[\begin{array}{ccc} \xi_{1}^{1}\left(t\right) & \ldots & \xi_{1}^{N}\left(t\right)\\ \xi_{2}^{1}\left(t\right) & \ldots & \xi_{2}^{N}\left(t\right) \end{array}\right]$$

be our estimate of the parameter θ and $V_{L}\left(t\right)$ be our estimate for $V_{R}$. We will design a learning algorithm to achieve c convergence of ξ(t) and $V_{L}\left(t\right)$ to the true values e.g., ξ(t)→θ and $V_{L}\left(t\right)\to V_{R}$ as t→∞.

### 2.3 Bounded Cost Path Planning

Our goal is to find a path connecting the vehicle current position $x_{0}$ to the final position $x_{f}$ that results in total travel time less than an upper bound C. In practice, the planning and replanning process of AUV happen over long intervals, in order to avoid increased computation cost. Hence we assume that the estimated parameters have converged to their true value counterparts before the planning process. There may be more than one path satisfying the bounded cost constraint. Thus we formulate the following optimization problem, in which the decision variable is the vehicle’s heading angle, $\psi_{C}\left(t\right)$. The optimization problem is to find the decision variable that is most likely to satisfy the bounded cost constraint:

$$\begin{array}{l} \underset{\psi_{C}\left(t\right)\in \left[-\pi ,\pi \right]}{\max}\mathrm{Pr}\left(T\left(\psi_{C}\left(t\right)\right)<C\right)\\ s.t.\;\;\;\;\;\dot{x}=V_{R}\Psi_{C}\left(t\right)+\theta \phi \left(x\right),\\ x\left(T_{0}\right)=x_{0},\\ x\left(T_{0}+T\left(\psi_{C}\left(t\right)\right)\right)=x_{f}. \end{array}$$ (4)

where the total travel time to start from the initial position $x_{0}$ to reach the destination position $x_{f}$ under the control signal $\psi_{C}\left(t\right)$ is denoted as $T\left(\psi_{C}\left(t\right)\right)$.

## 3 Flow Field Estimation

First let us describe Algorithm 1. We derive the spatial basis function and the initialized flow model parameters by solving eq. 2 using the K-means algorithm. Since this optimization depends on both spatial and temporal variance of the flow field, solving this problem can be computationally expensive. To simplify this problem, instead of optimizing the difference between the time-varying flow forecast and the partitioned flow field, as described in eq. 2, we optimize the difference between the time-averaged flow forecast and the partitioned flow field:

$$\underset{\phi \left(x\right),{\left\{\nu_{i}\right\}}_{i\in I_{R}}}{\min}J^{\prime }=\underset{i\in I_{R}}{\sum }\sum_{x\in {ℛ}_{i}}{\text{dist}}^{2}\left(\bar{y}\left(x\right),{\left[{\bar{r}}_{i}^{T},\nu_{i}^{T}\right]}^{T}\right)$$ (5)

where $\bar{y}\left(x\right)={\left[x^{T},\frac{1}{T_{f}-T_{0}}\underset{t\in [T_{0},T_{f}]}{\sum }F_{0}{\left(x,t\right)}^{T}\right]}^{T}$ denote the time averaged flow observation at position x.

**ALGORITHM 1:** Flow Field Partition Algorithm

**Data:** flow field observations $\left\{y\left(x_{i},t\right)\right\},i\in \left[1,m\right],t\in \left[T_{0},T_{f}\right]$, flow partition error threshold ϵ
**Output:** Spatial basis function $\phi \left(x_{i}\right)$, piece-wise constant flow speed of cells $\nu_{j},j\in \left[1,k\right]$
${\bar{F}}_{0}\left(x_{i}\right)=\frac{1}{T_{f}-T_{0}}\underset{t\in \left[T_{0},T_{f}\right]}{\sum }y\left(x_{i},t\right)$, ${\bar{y}}_{i}={\left[x_{i}^{T},{\bar{F}}_{0}{\left(x_{i}\right)}^{T}\right]}^{T}$
Initialize cluster number k=1, ${\bar{r}}_{1}=\frac{1}{m}\overset{m}{\underset{i=1}{\sum }}x_{i},\nu_{1}=\frac{1}{m}\overset{m}{\underset{i=1}{\sum }}{\bar{F}}_{0}\left(x_{i}\right)$
Compute δF using eq. 6
**while** δF>ε **do**
k=k+1 Randomly initialize cluster centroids
**while** not converges **do**
For all i∈[1,m], set $c_{i}=\underset{j}{\text{arg\textbackslash{}newspace}\;\min}{\;\text{dist}}^{2}\left({\bar{y}}_{i}{,\left[{\bar{r}}_{j}^{T},\nu_{j}^{T}\right]}^{T}\right)$
For all j∈[1,k], set ${\bar{r}}_{j}=\frac{\sum_{i}1\left(c_{i}=j\right)x_{i}}{\sum_{i}1\left(c_{i}=j\right)}$, $\nu_{j}=\frac{\sum_{i}1\left(c_{i}=j\right){\bar{F}}_{0}\left(x_{i}\right)}{\sum_{i}1\left(c_{i}=j\right)}$
Compute δF using eq. 6
**end**
**end**
For all i∈[1,m], $\phi \left(x_{i}\right)={\left[\begin{array}{ccc} 1\left\{c_{i}=1\right\} & \ldots & 1\left\{c_{i}=k\right\} \end{array}\right]}^{T}$
For all j∈[1,k], ${ℛ}_{j}=\cup_{i}1\left\{c_{i}=j\right\}x_{i}$

To implement the K-means algorithm, we start by randomly selecting k cell centroids, and then use Lloyd iterations to solve the optimization problem. The Lloyd iteration contains two steps, first assign the points that are closest to a centroid to that centroid, and then recompute the cell centroid. These two steps are repeated until cell membership no longer changes. The K-means algorithm requires proper selection of the number of partitioned cells, k, the choice of which affects the path planning performance and flow modeling quality. If the field is divided into too many regions, then it results in a complicated flow structure and potentially increases the computational cost of path planning. On the other hand, dividing the field into too few regions may result in a large error between the true flow field and the modeled flow field, which potentially leads to significant path planning error. Therefore, we introduce an iterative K-means algorithm that can guarantee a bounded flow field partition error, and at the same time utilize the smallest number of partition regions.

Let ${\bar{F}}_{0}:D\to {\mathbb{R}}^{2}$ denote the time-averaged flow field over the time interval $\left[T_{0},T_{f}\right]$, and $\nu_{i}$ is the uniform flow velocity in ${ℛ}_{i}$. Define the flow field partition error as:

$$\delta F=\underset{i\in I_{R}}{\max}\underset{x\in {ℛ}_{i}}{\max}\left\|{\bar{F}}_{0}\left(x\right)-\nu_{i}\right\|.$$ (6)

Given an initialized k, we will iteratively perform the K-means algorithm (lines 8, 9), and check if the flow partition error satisfies δF<ε, where ϵ is a pre-defined upper bound on the flow partitioning error. If this condition is satisfied, then the current k is designated for partitioning; otherwise, the number of cells is increased by 1, and we recompute the solution to eq. 5 using K-means method.

**ALGORITHM 2:** Flow Estimation Algorithm

**Data:** Vehicle trajectory x(t), estimated vehicle trajectory z(t), heading angle $\Psi_{C}\left(t\right)$, initial estimated parameter $\bar{\xi }\left(0\right)$, initial estimated speed $V_{L}\left(0\right)$
**Output:** Estimated parameter $\bar{\xi }\left(t+1\right)$, estimated flow parameter ξ(t+1), estimated vehicle speed $V_{L}\left(t+1\right)$
**while** t< ending time **do**
e(t)=x(t)−z(t)
Update β(t) using eq. 9
Update $\bar{\xi }$ and $V_{L}\left(t\right)$ using eq. 11
Update z(t) by integrating eq. 7
**end**

Now let us explain Algorithm 2. An estimate z(t) for the vehicle trajectory can be computed by integrating

$$\dot{z}=\xi \left(t\right)\phi \left(z\right)+V_{L}\left(t\right)\Psi_{C}+\beta \left(t\right),$$ (7)

where $\beta \left(t\right)\in {\mathbb{R}}^{2}$ is introduced as a learning injection parameter. The term e=x−z is the controlled Lagrangian Localization error (CLLE, Cho and Zhang, 2016; Cho et al., 2021), which describes how much the actual trajectory deviates from the estimated trajectory. A learning algorithm will then compute $\beta \left(t\right),\xi \left(t\right),V_{L}\left(t\right)$ so that the CLLE can be reduced.

The CLLE dynamics can be derived from eqs 7, 1.

$$\dot{e}=\dot{x}-\dot{z}=\theta \phi \left(x\right)-\xi \left(t\right)\phi \left(z\right)+\left(V_{R}-V_{L}\left(t\right)\right)\Psi_{c}-\beta \left(t\right).$$ (8)

We design the learning parameter injection as

β(t)=ξ(t)ϕ(x)−ξ(t)ϕ(z)+Ke, (9)

and hence the CLLE dynamics becomes

$$\dot{e}=-\text{Ke}+\left(\theta -\xi \left(t\right)\right)\phi \left(x\right)+\left(V_{R}-V_{L}\left(t\right)\right)\Psi_{c}.$$ (10)

The learning algorithm updates parameters ξ(t) and $V_{L}\left(t\right)$ so that the CLLE converges to zero. Let $\bar{\xi }\left(t\right)={\left[\xi_{1}^{1}\left(t\right),\ldots ,\xi_{1}^{N}\left(t\right),\xi_{2}^{1}\left(t\right),\ldots ,\xi_{2}^{N}\left(t\right)\right]}^{T},\bar{\theta }={\left[\theta_{1}^{1},\ldots ,\theta_{1}^{N},\theta_{2}^{1},\ldots ,\theta_{2}^{N}\right]}^{T}$, and $e⊗\phi ={\left[e_{1}\phi^{1},\ldots ,e_{1}\phi^{N},e_{2}\phi^{1},\ldots ,e_{2}\phi^{N}\right]}^{T}$, where ⊗ is the Kronecker product. We design the updating rules for parameter estimation as follows,

$$\begin{array}{l} \dot{\bar{\xi }}\left(t\right)=\text{ρe}⊗\phi \left(x\right)\\ {\dot{V}}_{L}\left(t\right)={\text{ρe}}^{T}\Psi_{c}. \end{array}$$ (11)

These rules are then used in Algorithm 2.

**ALGORITHM 3:** Bounded Cost Search in Piece-wise Constant Flow Field

**Data:** Start and goal node s, g, start and goal position $x_{0}$, $x_{f}$, travel cost upper bound C, graph G
**Output:** Optimal heading angle $\Psi_{C}$
PTS (s,g,C,G) →P *Step 1: Find the optimal cell sequence
MEJ $\left(P,x_{0},x_{f}\right)\to \Psi_{C}$ *Step 2: Find the optimal heading angle
**Function** PTS a(s,g,C,G)
Initialization. g(n)=∞,h(n)=∞,K(n)=∞,∀n∈G
s→{CLOSED}
Set the heuristics and estimated cost-of-arrival of s
(OPEN, CLOSED) = Expand (s,OPEN,CLOSED,G)
**while** OPEN is not empty **do**
**if** g∈ CLOSED **then**
Backtracking (s,g) →α
**end**
$v=\underset{n\in \text{OPEN}}{\arg\;\;\min\;\;}K\left(n\right)$
(OPEN, CLOSED) = Expand (v, OPEN, CLOSED)
**end**
**return** α
**Function** Epand v, OPEN, CLOSED, G
{OPEN}∖v→{OPEN}
$v\cup^{\text{​}}\left\{\text{CLOSED}\right\}\to \left\{\text{CLOSED}\right\}$
Find adjacent nodes ${\left\{n_{i}\right\}}_{i=1}^{m}$ to v in G
**for** i=1 to m **do**
Compute minimum branch cost $w^{*}\left(v,n_{i}\right)$ by solving eq. 17
**if** $w^{*}\left(v,n_{i}\right)+g\left(v\right)<g\left(n_{i}\right)$ $n_{i}$ **then**
predecessor = v
$\left\{\text{OPEN}\right\}\cup^{\text{​}}n_{i}\to \left\{\text{OPEN}\right\}$
Compute $h\left(n_{i}\right)$ using eq. 18
$g\left(n_{i}\right)=g\left(v\right)+w^{*}\left(v,n_{i}\right)$
Update $K\left(n_{i}\right)$ using eq. 19
**end**
**end**
**return** OPEN, CLOSED
**Function** Backtrack (s,g)
g→P
**while** s∉{P} **do**
v=P(end)
v.predecessor $\cup^{\text{​}}P\to P$
**end**
**return** P
**Function** MEJ $\left(P,x_{0},x_{f}\right)$
Initialize junction set $\gamma^{0}$ on the boundary curves of the cell sequence P
Compute junction positions by solving eq. 20
Compute heading angle from junction positions by eq. 21
**return** $\Psi_{C}\left(t\right)$

## 4 Bounded Cost Path Planning

Given the piece-wise constant flow model described in eq. 3, the domain is divided into a finite number of regions ${\left\{{ℛ}_{i}\right\}}_{i\in I_{R}}$. Thus, all possible trajectories cross a sequence of cells of uniform flow, and finally reach the goal position. Since the vehicle moves in constant speed, and the flow in one cell is uniform and constant, the vehicle’s optimal heading angle in each cell should be constant, and the vehicle path in each cell is a straight line. We define junction points as the position where the path intersects with cell boundaries. Below we show that in each cell, due to the time invariance of the flow field, solving for the heading angle is equivalent to solving for the junction points of a path.

Let $\gamma_{1},\gamma_{2}$ denote two junction points on two different boundary curves of the same cell ${ℛ}_{i}$. Since the vehicle moves at constant speed, the total vehicle speed $V_{R}\Psi_{C}+\theta^{i}$ must be in the same direction as the segment of the path,

$$\frac{\gamma_{2}-\gamma_{1}}{\left|\left|\gamma_{2}-\gamma_{1}\right|\right|}=\frac{V_{R}\Psi_{C}+\theta^{i}}{\left|\left|V_{R}\Psi_{C}+\theta^{i}\right|\right|}.$$ (12)

From eq. 12, we can represent the vehicle’s heading angle as a function of the junction points,

$$\Psi_{C}=\frac{1}{V_{R}}\left(\frac{\gamma_{2}-\gamma_{1}}{\left|\left|\gamma_{2}-\gamma_{1}\right|\right|}\sqrt{V_{R}^{2}+2V_{R}{\left(\theta^{i}\right)}^{T}\Psi_{C}+{\left|\left|\theta^{i}\right|\right|}^{2}}-\theta^{i}\right).$$ (13)

The vehicle’s travel time in ${ℛ}_{i}$, denoted as τ, can be computed given the junction points and the vehicle’s heading angle,

$$\tau \left(\gamma_{1},\gamma_{2},\theta^{i}\right)=\frac{\left|\left|\gamma_{2}-\gamma_{1}\right|\right|}{\left|\left|V_{R}\Psi_{C}+\theta^{i}\right|\right|}.$$ (14)

Combining eq. 14 and eq. 12 we can write the travel time in cell ${ℛ}_{i}$ as a function dependent only on the junction points,

$$\tau \left(\gamma_{1},\gamma_{2},\theta^{i}\right)=\frac{1}{{\left|\left|\theta^{i}\right|\right|}^{2}-V_{R}^{2}}\left({\left(\gamma_{2}-\gamma_{1}\right)}^{T}\theta^{i}-\sqrt{{\left({\left(\gamma_{2}-\gamma_{1}\right)}^{T}\theta^{i}\right)}^{2}+{\left\|\gamma_{2}-\gamma_{1}\right\|}^{2}\left(V_{R}^{2}-{\left|\left|\theta^{i}\right|\right|}^{2}\right)}\right).$$ (15)

eq. 15 describes the travel time in one single cell. Based on the discussion of the one cell case, next we talk about solving eq. 4 across multiple cells.

Let $\gamma_{1},\gamma_{2},\ldots ,\gamma_{n}$ denote the chain of junctions position, and $p_{1},p_{2},\ldots ,p_{n+1}$ represent the index of the sequence of cells that the path crosses. The planning problem eq. 4 can be transformed into the following mixed integer optimization problem, where the decision variables are the cell sequence and the junctions position,

$$\begin{array}{l} \underset{{\left\{\gamma_{i}\right\}}_{i=1}^{n},{\left\{p_{i}\right\}}_{i=1}^{n+1}}{\max}\mathrm{Pr}\left(\tau \left(x_{s},\gamma_{1},\theta^{p_{1}}\right)+\overset{n-1}{\underset{i=1}{\sum }}\tau \left(\gamma_{i},\gamma_{i+1},\theta^{p_{i}}\right)+\tau \left(\gamma_{n},x_{f},\theta^{p_{n+1}}\right)<C\right),\\ s.t.\;f_{p_{i},p_{i+1}}\left(\gamma_{i}\right)=0,\forall i\in \left[1,n\right]. \end{array}$$ (16)

Now let us explain the proposed solution to eq. 16. The solution is presented in Algorithm 3. We propose a bounded cost path planning algorithm that solves for the path that is most likely to satisfy the bounded cost constraint. The solution contains two steps, the first step is solving for the optimal sequence of cells that is most likely to result in a bounded cost path, in the discretized flow map described by the piece-wise constant flow cells. In this step, the junction positions are unknown. An informed graph search method is used for the first step of the proposed solution. The second step is optimizing junction positions on the boundaries of the optimal cell sequence.

**FIGURE 1:**
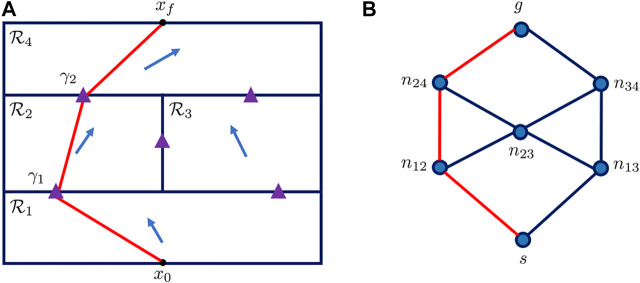
**(A)** Partitioned cells in the domain. On each boundary of two adjacent cells there is a candidate junction point, represented as the purple triangle **(B)** Graph representation of the workspace. The vertices represent the candidate junctions, while the edges are the path segment between the adjacent junctions. The red line on both of the plots represent the same example path.

The proposed solution is presented in Algorithm 3. First we describe the first step of our solution. Consider having a candidate junction on boundaries of all cells. Two junctions $\gamma_{i}$ and $\gamma_{j}$ are defined as adjacent if $f_{p,q}\left(\gamma_{i}\right)=0,f_{r,s}\left(\gamma_{j}\right)=0,\left\{p,q\right\}\cap^{\text{​}}\left\{r,s\right\}\neq \emptyset$, indicating that the two junctions are on different boundaries of the same cell. Two adjacent junctions are connected by an edge. A non-directed graph G can be formed with the vertices being all the candidate junctions in the domain, and the edges being the path segment between the adjacent candidate junctions. Let $n_{i,j}$ describe the node that corresponds to the junction on boundary curve between ${ℛ}_{i}$ and ${ℛ}_{j}$, and let s and g denote the node corresponding to the starting and final position. Then in this context, a path Γ from the starting position $x_{0}$ to the final position $x_{f}$, crossing the cells ${ℛ}_{p_{1}},\ldots ,{ℛ}_{p_{n+1}}$ in sequence can be represented by a sequence of nodes $s\to n_{p_{1}p_{2}}\to n_{p_{2}p_{3}}\to \ldots \to n_{p_{n}p_{n+1}}\to g$ on the graph. Figure 1 is an example of the graph representation of the workspace, in which case the flow field is partitioned into 4 cells.

The branch cost of the graph is defined as the travel time from one junction to another adjacent junction. The travel time can be computed by eq. 15 if the two junction positions are known. However, since the junction positions are unknown when optimizing the cell sequence, the branch cost of the graph cannot be explicitly computed. Hence we introduced the following assumption:

Assumption 4.1 : We assume that the branch cost for all edges in ℰ is a random variable, with a known minimum value.

Remark 4.1 : Even though the branch cost is unknown, its minimum value can be computed, since the branch cost eq. 15 is convex with respect to $\gamma_{2}-\gamma_{1}$ (Soulignac, 2011).

We solve for the minimum cost of all edges in the graph, denoted as $w_{ij,jk}^{*}$ by solving the following constrained optimization problem, using the interior-point method. (Kim et al., 2007),

$$\begin{array}{l} \underset{\gamma_{1},\gamma_{2}}{\min}\tau \left(\gamma_{1},\gamma_{2},\theta^{j}\right)\\ s.t.\;\;\;\;\;\;f_{ij}\left(\gamma_{1}\right)=0,f_{jk}\left(\gamma_{2}\right)=0. \end{array}$$ (17)

The informed graph search method we propose is an extension to a class of graph search algorithms called potential search (PTS) (Stern et al., 2014). The PTS algorithms can be viewed as modifications to the celebrated A* algorithm for path planning (Hart et al., 1968). To determine which nodes should be searched, the algorithms maintain an OPEN list and a CLOSED list. A graph node is labeled NEW if it has not been searched by the algorithm. The OPEN list contains all the nodes that are searched, but still have a NEW neighbor. The CLOSED list consists of all the nodes that have been accessed by the search algorithm.

To determine which cells should be searched first by the algorithm, the algorithm computes the cost-of-arrival, which is the minimal cost of going from the starting node s to an arbitrary node n, and cost-to-go, which is the minimal cost of going from n to the goal point g. Let $g^{*}\left(n\right)$ denote the actual cost-of-arrival, and let $h^{*}\left(n\right)$ denote the actual cost-to-go of a node n. Since the actual cost-to-go is unknown during the search, a heuristic cost $h\left(n\right)\leq h^{*}\left(n\right)$, is usually used by the search algorithm. The A* search algorithm sort the OPEN list according to the value of $g^{*}\left(n\right)+h\left(n\right)$. The node with lowest value is searched first.

In our problem, the following estimated cost-to-go is used to guide the search:

$$h\left(n\right)=\frac{\underset{\gamma_{n}}{\min}\left\|x_{f}-\gamma_{n}\right\|}{V_{R}+\underset{i\in I_{R}}{\max}\left\|\theta^{i}\right\|}.$$ (18)

The heuristic function defined in eq. 18 is the travel time of the vehicle traveling in the most favorable flow condition, reaching goal position from the junction position that is closest to the goal. Hence, $h\left(n\right)\leq h^{*}\left(n\right)$, which is required by A* search. However, in our problem, the branch cost is unknown. The exact value of actual cost-of-arrival cannot be computed during the search process, which is different from a typical path planning problem that can be solved by A* or PTS method. Hence we introduce the estimated cost-of-arrival, denoted as g(n). The estimated cost-of-arrival is computed by summation of the minimum branch cost along the path, thus $g\left(n\right)\leq g^{*}\left(n\right)$.

The goal of A* search is to find the path with minimum cost. In our problem, due to the uncertainties in the branch cost, this goal is overly ambitious. Hence our problem formulation eq. 16 aims to find a path with bounded cost. We define a potential function described as follows:

Definition 4.1 : The potential of a node n, denoted as PT(n), is $\mathrm{Pr}\left(h^{*}\left(n\right)+g^{*}\left(n\right)<C\right)$.

The potential function characterizes the probability that a node is on a path that satisfies the bounded cost constraint. Nodes with high potential have higher probability to be part of the desired path. However, the exact potential of nodes cannot be computed or compared, since both $h^{*}\left(n\right)$ is unknown before the optimal path is found. Therefore, PTS algorithms usually design a key function to determine the nodes that need to be searched at each step of the graph search. Nodes in the OPEN list are sorted by the key function value instead of $g^{*}\left(n\right)+h\left(n\right)$, which is the main difference between the PTS algorithms and the A* method. Various key functions have been proposed for different path planning problems with bounded cost (Thayer et al., 2012; Stern et al., 2014, Stern et al., 2011).

 One significant contribution of this paper is in extending the PTS method to solving bounded cost problems with uncertain branch cost, by introducing a new form of key function $K\left(n\right)\in {\mathbb{R}}_{\geq 0}$ as

$$K\left(n\right)=\{\begin{array}{cc} \frac{h\left(n\right)g\left(n\right)}{{\left(C-h\left(n\right)-g\left(n\right)\right)}^{2}}, & \text{if}\;h\left(n\right)+g\left(n\right)<C\\ \infty , & \text{otherwise} \end{array}.$$ (19)

K(n) is an indication of the probability of the node n being on a path that satisfies the bounded cost constraint. Nodes with lower key function value have a higher probability of being on a path satisfying the bounded cost constraint. The intuition is that, if h(n)+g(n)<C, the key function value increases if either h(n) or g(n) is larger. In this case, the estimated cost h(n)+g(n) increases, and will be closer to C, then it is less likely that the true cost satisfies the bounded cost constraint, and the node n is less likely to be on a feasible path. If h(n)+g(n)≥C, then n cannot be on a path satisfying the bounded cost constraint, since $h^{*}\left(n\right)+g^{*}\left(n\right)\geq h\left(n\right)+g\left(n\right)\geq C$. In this case, the key function is set as positive infinity.

The PTS with our new key function is then applied to search for the optimal cell in Algorithm 3. The only difference between our PTS and A* is that the total cost used by A* to sort the OPEN list is replaced by the key function, as shown in line 13. Similar to A*, The search algorithm consists of two processes: the expansion process and the backtracking process. During the iterative expansion process, the algorithm orders the nodes in the OPEN set according to the key function value, and inserts the node with the lowest key function value to the CLOSED set (lines 19, 20). Neighbors of this node and their key function values are updated if the neighboring nodes can be reached with a lower cost through the current node (lines 26, 28, 29). The propagation continues until the OPEN list is depleted, or the goal node is in the OPEN set. Starting from the goal position, the backtracking process searches for the predecessor of the last node in the path set and add it to the path, until the starting node is included in the path (lines 37, 38).

The PTS algorithm fulfills step one of the bounded cost path planning solution. We have found the vector of indices ${\left\{p_{i}\right\}}_{i=1}^{n+1}$, which indicates the optimal indices that is most likely to result in a bounded cost path. In step two, we find the optimal junction positions that leads to the minimum total cost. Given the optimal cell sequence, the problem eq. 16 converts to an optimization problem depending on the junction positions ${\left\{\gamma_{i}\right\}}_{i=1}^{n}$ in all cells,

$$\begin{array}{l} \underset{{\left\{\gamma_{i}\right\}}_{i=1}^{n}}{\min}\tau \left(x_{0},\gamma_{1},\theta^{p_{1}}\right)+\overset{n}{\underset{i=1}{\sum }}\tau \left(\gamma_{i},\gamma_{i+1},\theta^{p_{i}}\right)+\tau \left(\gamma_{n},x_{f},\theta^{p_{n+1}}\right)\\ s.t.\;f_{p_{i},p_{i+1}}\left(\gamma_{i}\right)=0. \end{array}$$ (20)

This optimization problem is solved by the interior-point method.

The optimal heading angle can be computed from the junction positions using eq. 12. In each cell of the sequence ${\left\{p_{i}\right\}}_{i=1}^{n}$, given the optimal junction position $\gamma_{i+1}$ and $\gamma_{i}$, the heading angle in cell ${ℛ}_{p_{i}}$ can be derived by

$$\Psi_{C}=\frac{1}{V_{R}}\left(\frac{\gamma_{i+1}-\gamma_{i}}{\left|\left|\gamma_{i+1}-\gamma_{i}\right|\right|}\sqrt{{V_{R}^{2}+2V_{R}\left(\theta^{p_{i}}\right)}^{T}\Psi_{C}+{\left\|\theta^{p_{i}}\right\|}^{2}}-\theta^{p_{i}}\right).$$ (21)

## 5 Theoretical Justification

In this section, we give theoretical justification to the proposed data-driven flow modeling and bounded cost path planning method.

### 5.1 Data-Driven Flow Modeling

Algorithm 1 can be theoretically justified by proving that the optimal solution to eq. 5 is the optimal solution to eq. 2. We also prove that Algorithm 2 achieves error convergence and parameter convergence, indicating that the estimated trajectory converges to the actual trajectory, and the estimated parameter converges to the true values.

Lemma 5.1: The optimal flow partition derived by solving eq. 2 is the same as the optimal flow partition derived from eq. 5.

 PROOF: Let $\delta y\left(x,t\right)=y\left(x,t\right)-\bar{y}\left(x\right)$. Since $\overset{T_{f}}{\underset{t=T_{0}}{\sum }}\delta y\left(x,t\right)=\overset{T_{f}}{\underset{t=T_{0}}{\sum }}y\left(x,t\right)-\left(T_{f}-T_{0}\right)\bar{y}\left(x\right)=0$, the following equality holds

$$\begin{array}{l} J=\overset{N}{\underset{i=1}{\sum }}\underset{x\in {ℛ}_{i}}{\sum }\sum_{t=T_{0}}^{T_{f}}{\text{dist}}^{2}\left(y\left(x,t\right),\mu_{i}\right)\\ \;\;\;\;\;=\overset{N}{\underset{i=1}{\sum }}\underset{x\in {ℛ}_{i}}{\sum }\sum_{t=T_{0}}^{T_{f}}{\text{dist}}^{2}\left(\bar{y}\left(x\right)+\delta y\left(x,t\right),\mu_{i}\right)\\ \;\;\;\;\;=\overset{N}{\underset{i=1}{\sum }}\sum_{x\in {ℛ}_{i}}\left[\left(T_{f}-T_{0}\right){\text{dist}}^{2}\left(\bar{y}\left(x\right),\mu_{i}\right)+\sum_{t=T_{0}}^{T_{f}}{\text{dist}}^{2}\left(y\left(x,t\right),\bar{y}\left(x\right)\right)\right]. \end{array}$$

The second term in J represents the temporal variation of flow speed on one grid point, which does not change with respect to the partitioning of the flow field. Hence $\underset{\phi \left(x\right),\nu }{\arg\;\;\min}J=\underset{\phi \left(x\right),\nu }{\arg\;\min}J\prime$, where J′ is defined in eq. 5. Thus the optimal solution of eq. 2 equals the optimal solution of eq. 5, which implies that the optimal flow partition of the time-varying flow field is equivalent to the optimal flow partition of the time-invariant flow field, computed by taking the time-average of the flow field observations, as described in line 1, Algorithm 1.

 Next we will prove that under Assumption 2.3, the estimated trajectory converges to the actual trajectory, and that the estimated parameter converges to the true value using adaptive control theory. In order to prove convergence, the persistent excitation condition must be demonstrated, and is given below.

Definition 5.2: (Sastry and Bodson, 1994; Khalil, 1996) A vector signal u is persistently exciting if there exist positive constants $\kappa_{1},\kappa_{2}$, and T such that $\kappa_{2}I\geq \overset{t+T}{\underset{t}{\int }}u\left(\tau \right)u^{T}\left(\tau \right)d\tau \geq \kappa_{1}I\;\forall t$.

Let ${\tilde{\phi }}_{1}=\left[\begin{array}{ccc} \phi^{1} & \ldots & \phi^{N}\\ 0 & 0 & 0 \end{array}\right]$ and ${\tilde{\phi }}_{2}=\left[\begin{array}{ccc} 0 & 0 & 0\\ \phi_{1} & \ldots & \phi^{N} \end{array}\right]$. Let $w={\left[{\tilde{\phi }}_{1},{\tilde{\phi }}_{2},\Psi_{C}\right]}^{T}\in {\mathbb{R}}^{\left(2N+1\right)\times 2}$, which is the input signal to eq. 24. We can construct a matrix $W\left(t\right)\in {\mathbb{R}}^{\left(2N+1\right)\times \left(2N+1\right)}$ as follows

$$W\left(t\right)={\int^{\text{​}}}_{t}^{t+T}\left[\begin{array}{cccccccc} \phi^{1}\phi^{1} & \phi^{1}\phi^{2} & \ldots & \phi^{1}\phi^{N} & 0 & \ldots & 0 & \phi^{1}\cos\psi_{c}\\ \phi^{2}\phi^{1} & \phi^{2}\phi^{2} & \ldots & \phi^{2}\phi^{N} & 0 & \ldots & 0 & \phi^{2}\cos\psi_{c}\\ \vdots & \ddots & \vdots & \vdots & \vdots & \ddots & \vdots & \vdots \\ \phi^{N}\phi^{1} & \phi^{N}\phi^{2} & \ldots & \phi^{N}\phi^{N} & 0 & \ldots & 0 & \phi^{N}\cos\psi_{c}\\ 0 & 0 & \ldots & 0 & \phi^{1}\phi^{1} & \ldots & \phi^{1}\phi^{N} & \phi^{1}\sin\psi_{c}\\ 0 & 0 & \ldots & 0 & \phi^{2}\phi^{1} & \ldots & \phi^{2}\phi^{N} & \phi^{2}\sin\psi_{c}\\ \vdots & \vdots & \ddots & \vdots & \vdots & \ddots & \vdots & \vdots \\ 0 & 0 & \ldots & 0 & \phi^{N}\phi^{1} & \ldots & \phi^{N}\phi^{N} & \phi^{N}\sin\psi_{c}\\ \phi^{1}\cos\psi_{c} & \phi^{2}\cos\psi_{c} & \ldots & \phi^{N}\cos\psi_{c} & \phi^{1}\sin\psi_{c} & \ldots & \phi^{N}\sin\psi_{c} & 1 \end{array}\right]d\tau .$$ (22)

The persistent excitation condition is critical to prove the convergence of parameters (Narendra and Annaswamy, 1989). When W(t) is singular the estimation errors of parameters may not converge to zero. The persistent excitation condition requires that the trajectories traveled by the robot to spread over all the partitioned cells, as stated in the following Lemma.

Lemma 5.3: The signal vector w is persistently exciting if the vehicle visits all the partitioned cells.

 PROOF: Since the partitioned cells do not overlap with each other, for ∀τ, x(τ) can only be in one cell. Hence for all $i,j\in I_{R}$,

$$\phi^{i}\left(x\left(\tau \right)\right)\phi^{j}\left(x\left(\tau \right)\right)=11\{i=j\}=\{\begin{array}{cc} 1 & \text{if}\;i=j\\ 0 & \text{otherwise} \end{array}.$$

Thus W(t) can be simplified to

$$W\left(t\right)={\int^{\text{​}}}_{t}^{t+T}\left[\begin{array}{ccccccc} \phi^{1}\phi^{1} & & & & & & \phi^{1}\cos\psi_{c}\\ & \ddots & & & 0 & & \vdots \\ & & \phi^{N}\phi^{N} & & & & \phi^{N}\cos\psi_{c}\\ & & & \phi^{1}\phi^{1} & & & \phi^{1}\sin\psi_{c}\\ & 0 & & & \ddots & & \vdots \\ & & & & & \phi^{N}\phi^{N} & \phi^{N}\sin\psi_{c}\\ \phi^{1}\cos\psi_{c} & \ldots & \phi^{N}\cos\psi_{c} & \phi^{1}\sin\psi_{c} & \ldots & \phi^{N}\sin\psi_{c} & 1 \end{array}\right]d\tau .$$

If $\forall i,\exists \tau \in \left[t,t+T\right],s.t.\;\phi^{i}\left(x\left(\tau \right)\right)=1$, meaning that the vehicle visits all cell during time [t,t+T], then W(t) is full rank, and hence w is persistently exciting.

The persistent excitation condition must be satisfied in order to have the flow parameters of all the cells and vehicle speed estimation converge to the true value. The persistent excitation condition requires the vehicle to visit all the partitioned regions. If this condition is not satisfied, not all flow parameters in the partitioned cells can be accurately estimated. We will further address this condition in the simulation and experimental result section.

The convergence of CLLE is presented as follows.

Theorem 5.4: Under the updating law eq. 11, CLLE converges to zero when time goes to infinity.

 PROOF: Consider the following Lyapunov function,

$$V\left(e,\bar{\xi },V_{L}\right)=\frac{1}{2}\left(e^{T}e+\frac{1}{\rho }{\left(\bar{\theta }-\bar{\xi }\left(t\right)\right)}^{T}\left(\bar{\theta }-\bar{\xi }\left(t\right)\right)+\frac{1}{\rho }{\left(V_{R}-V_{L}\left(t\right)\right)}^{2}\right).$$

Since $e^{T}\left(\theta -\xi \left(t\right)\right)\phi \left(x\right)=\left(\bar{\theta }-\bar{\xi }\left(t\right)\right)e⊗\phi \left(x\right)$, the derivative of V is

$$\begin{array}{l} \dot{V}={\left(-\text{Ke}+\left(\theta -\xi \right)\phi \left(x\right)+\left(V_{R}-V_{L}\left(t\right)\right)\Psi_{C}\right)}^{T}e+\frac{1}{\rho }\left(-\text{ρe}⊗\phi \left(x\right)\right)\left(\bar{\theta }-\bar{\xi }\right)\\ \;\;\;\;\;+\frac{1}{\rho }\left(V_{R}-V_{L}\left(t\right)\right)\left(-{\text{ρe}}^{T}\Psi_{C}\right)\\ \;\;\;\;\;=e^{T}\text{Ke}\leq 0. \end{array}$$

$\dot{V}$ is negative semi-definite, which implies that $e,\bar{\xi },V_{L}\left(t\right)$ are bounded. In addition, the second order time derivative of V is

$$\ddot{V}=-2e^{T}K\left(\left(\theta -\xi \left(t\right)\right)\phi \left(x\right)+\left(V_{R}-V_{L}\left(t\right)\right)\Psi_{C}-\text{Ke}\right).$$

Thus $\ddot{V}$ bounded, and hence $\dot{V}$ is uniformly continuous. Therefore $\lim_{t\to \infty }\dot{V}\left(t\right)=0$. Since K is a diagonal matrix, e(t)→0 as t→∞.

Theorem 5.5: Under the updating law eq. 11, if the vehicle visits all the partitioned cells, $\bar{\xi }\left(t\right)$ and $V_{L}\left(t\right)$ converges to $\bar{\theta }$ and $V_{R}$ respectively as time goes to infinity.

 PROOF: Let $\eta_{1}=\theta_{1}-\xi_{1}\left(t\right),\eta_{2}=\theta_{2}-\xi_{2}\left(t\right),\eta_{3}=V_{R}-V_{L}\left(t\right)$, then the CLLE dynamics can be written as

$$\dot{e}={\tilde{\phi }}_{1}\left(x\right)\eta_{1}+{\tilde{\phi }}_{2}\left(x\right)\eta_{2}+\eta_{3}\Psi_{C}-\text{Ke}.$$

We define a new state variable $X={\left[e^{T},\eta_{1}^{T},\eta_{2}^{T},\eta_{3}\right]}^{T}$, and an output variable Y=e, then the dynamics for the state variable and the output variable satisfy

$$\dot{X}=A\left(t\right)X,Y=\text{CX},$$

where $A\left(t\right)=\left[\begin{array}{cccc} -K & {\tilde{\phi }}_{1} & {\tilde{\phi }}_{2} & \Psi_{C}\\ -\rho {\tilde{\phi }}_{1} & 0 & 0 & 0\\ -\rho {\tilde{\phi }}_{2} & 0 & 0 & 0\\ -\rho \Psi_{C} & 0 & 0 & 0 \end{array}\right],C=\left[\begin{array}{cccc} I & 0 & 0 & 0 \end{array}\right].$

Our goal is to show that the origin of $\dot{X}=A\left(t\right)X$ is uniformly asymptotically stable, which indicates that $\bar{\xi }$ converges to $\bar{\theta }$, and $V_{L}\left(t\right)$ converges to $V_{R}$. Let

$$P=\left[\begin{array}{cccc} \frac{1}{2}K^{-1} & 0 & 0 & 0\\ 0 & \frac{1}{2\rho }K^{-1} & 0 & 0\\ 0 & 0 & \frac{1}{2\rho }K^{-1} & 0\\ 0 & 0 & 0 & \frac{1}{2\rho }K^{-1} \end{array}\right].$$

There exists some $c_{1},c_{2}$ such that $c_{1}I\leq P\leq c_{2}I$, and there exists some constant 0<ν<1 such that

$$A{\left(t\right)}^{T}P+\text{PA}\left(t\right)+\dot{P}+{\text{νC}}^{T}C=\left(1-\nu \right)\left[\begin{array}{cccc} -I & 0 & 0 & 0\\ 0 & 0 & 0 & 0\\ 0 & 0 & 0 & 0\\ 0 & 0 & 0 & 0 \end{array}\right],$$

which is negative semi-definite.

Then by the Lyapunov theorem (Theorem 3.8.4 in (Ioannou and Sun, 1995)), $\dot{X}=A\left(t\right)X$ is uniformly asymptotically stable if we can prove that (C,A) is uniformly completely observable. First, we will find a bounded matrix L, and show that (C,A+LC) is uniformly completely observable. Then, this will lead to the conclusion that (C,A) is uniformly completely observable.

Let $L=\left[\begin{array}{c} 0\\ \rho {\tilde{\phi }}_{1}\\ \rho {\tilde{\phi }}_{2}\\ \rho \Psi_{C}^{T} \end{array}\right]$. Since $\Psi_{C}$ is uniformly bounded, and all elements in $\tilde{\phi }$ is either 0 or 1, L is uniformly bounded, and

$$A+\text{LC}=\left[\begin{array}{cccc} -K & {\tilde{\phi }}_{1} & {\tilde{\phi }}_{2} & \Psi_{C}\\ 0 & 0 & 0 & 0\\ 0 & 0 & 0 & 0\\ 0 & 0 & 0 & 0 \end{array}\right].$$

Thus, we now consider the observability of

$$\begin{array}{l} \dot{X}=\left(A+\text{LC}\right)X\\ Y=\text{CX}. \end{array}$$ (23)

Let $\eta ={\left[\eta_{1},\eta_{2},\eta_{3}\right]}^{T}$, then the system eq. 23 has the following form:

$$\begin{array}{cc} \dot{e} & =-\text{Ke}+w^{T}\eta \\ \dot{\eta } & =0\\ Y & =e \end{array}.$$ (24)

Due to the assumption that the vehicle visits all cells, by Lemma 5.3, w is persistently exciting. Let $\Phi \left(\tau \right)=\overset{\tau }{\underset{t}{\int }}e^{-K\left(\tau -\sigma \right)}w\left(\sigma \right)d\sigma$ be the output of eq. 24 given input w. Then Φ(τ) satisfies persistently exciting conditions because w(σ) is persistently exciting, and the transfer function of eq. 24, ${\left(sI+K\right)}^{-1}$ is stable, minimum phase, proper rational transfer function. Therefore, there exists constant $\kappa_{1},\kappa_{2},T_{0}>0$ such that $\kappa_{2}I\geq \frac{1}{T_{0}}\overset{t+T_{0}}{\underset{t}{\int }}\Phi \left(\tau \right)\Phi {\left(\tau \right)}^{T}d\tau \geq \kappa_{1}I,\forall t\geq 0$. By applying Lemma 4.8.4 in (Ioannou and Sun, 1995), we can conclude that the system of eq. 24 is uniformly completely observable. In other words, we have proved that (C,A+LC) is uniformly completely observable. By applying Lemma 4.8.1 in (Ioannou and Sun, 1995) the system (C,A) is uniformly completely observable. Therefore, the origin of $\dot{X}=\text{AX}$ is uniformly asymptotically stable, that is, X→0 as t→∞. This means that $\eta_{1},\eta_{2}$ and $\eta_{3}$ go to zeros, individually. Thus $\bar{\xi }$ and $V_{L}\left(t\right)$ converges to $\bar{\theta }$ and $V_{R}$, respectively.

### 5.2 Bounded Cost Path Planning

In this subsection, we prove that Algorithm 3 finds the optimal solution of eq. 4. Assumption 5.1 and Assumption 5.2 are required for the optimality proof.

Assumption 5.1: Consider any node not the starting node s or the goal node g, the estimated cost-of-arrival g(n) and the estimated cost-to-go h(n) are bounded below, $g\left(n\right)\geq g_{\min},h\left(n\right)\geq h_{\min}$, where $g_{\min}>0$ and $h_{\min}>0$.

Remark 5.1: For any node that is not the goal node, h(n) reaches its minimum when n is an adjacent node of g. Similarly, for any node that is not the start node, g(n) reaches its minimum when n is adjacent to s. Since the flow partition algorithm is performed over discrete grid points in D, size of the cells cannot be infinitely small. Therefore, h(n) can only be zero if the junction represented by the node n is sliding on the same boundary of the goal point, and g(n) can only be zero if the junction represented by the node n is sliding on the same boundary of the start point. However, by junction assignment, only one junction can be assigned on each boundary. Hence there exists $h_{\min}$ and $g_{\min}$ that bound h(n) and g(n) from below, and the lower bound cannot be infinitely small.

Let $H_{\max}=\max\left\{\frac{C}{h_{\min}},\frac{C}{g_{\min}}\right\}$. Consider ${\left\{X_{n}\right\}}_{n=1}^{N},{\left\{Y_{n}\right\}}_{n=1}^{N}$ to denote sequences of independent and identically distributed random variables uniformly distributed over $\left[1,H_{\max}\right]$. To prove optimality of the algorithm, we make assumptions on the statistical relationship between $h^{*}\left(n\right),h\left(n\right)$, and $g^{*}\left(n\right),g\left(n\right)$ as follows.

Assumption 5.2 : The true cost-to-go, $h^{*}\left(n\right)$ and the heuristic function h(n), as well as the true cost-of-arrival, $g^{*}\left(n\right)$ and estimated cost-of-arrival, g(n) both satisfy $h^{*}\left(n\right)=h\left(n\right)Y_{n},g^{*}\left(x\right)=g\left(n\right)X_{n}$.

Remark 5.2: Both $h^{*}\left(n\right)$ and $g^{*}\left(n\right)$ are summation of branch cost along the optimal path. Since the branch cost is the travel time between two adjacent junctions sliding on two boundaries, the branch cost of all edges must have both a lower bound and an upper bound. Hence both $h^{*}\left(n\right)$ and $g^{*}\left(n\right)$ are assumed to be a uniform distribution, with the minimum of it being h(n) and g(n), and the maximum being $h\left(n\right)H_{\max}$ and $g\left(n\right)H_{\max}$. In practice, the statistical model of $h^{*}\left(n\right)$ and $g^{*}\left(n\right)$ depends on the distribution of the flow field, and may not be uniform distribution in some flow cases. However, the following theoretical analysis can be adapted to other parameterization of the statistical model of $h^{*}\left(n\right)$ and $g^{*}\left(n\right)$.

We will show below that, by expanding the nodes with the lowest key function value without explicitly calculating the potential of nodes, the proposed algorithm expands the nodes with the highest potential value, and thus guarantees to find the optimal solution to eq. 4. Lemma 5.6 states that the key function is an equivalent evaluation of the potential value of nodes. Lemma 5.7 demonstrates that the optimal path can be equally defined by either the potential or the key function value of nodes. Finally, given the two Lemmas, we justify the optimality of the proposed algorithm, which is stated in Theorem 5.8.

Lemma 5.6: For all $n_{1},n_{2}\in G$, $\text{PT}\left(n_{1}\right)<\text{PT}\left(n_{2}\right)$ if and only if $K\left(n_{1}\right)>K\left(n_{2}\right)$.

 PROOF: To simplify notation, let $h_{1},h_{1}^{\text{*}},g_{1},g_{1}^{\text{*}}$ denote $h\left(n_{1}\right),h^{\text{*}}\left(n_{1}\right),g\left(n_{1}\right)$, and $g^{\text{*}}\left(n_{1}\right)$, respectively. The Lemma trivially holds in the cases where either $K\left(n_{1}\right)$ or $K\left(n_{2}\right)$ is infinity. Below we show that the Lemma holds in the case where both $K\left(n_{1}\right)$ and $K\left(n_{2}\right)$ are not infinity; equivalently, $h_{1}+g_{1}<C$ and $h_{2}+g_{2}<C$. Due to the i.i.d. distribution assumption stated in Assumption 5.2, $X_{1},X_{2}$ can be written as a single random variable uniformly distributed on $\left[1,H_{\text{max}}\right]$, and $Y_{1},Y_{2}$ also can be written as a single random variable uniformly distributed on $\left[1,H_{\text{max}}\right]$. Therefore, $\text{PT}\left(n_{1}\right)<\text{PT}\left(n_{2}\right)$ if and only if

$$\text{Pr}\left(h_{1}Y+g_{1}X<C\right)<Pr\left(h_{2}Y+g_{2}X<C\right).$$

**FIGURE 2:**
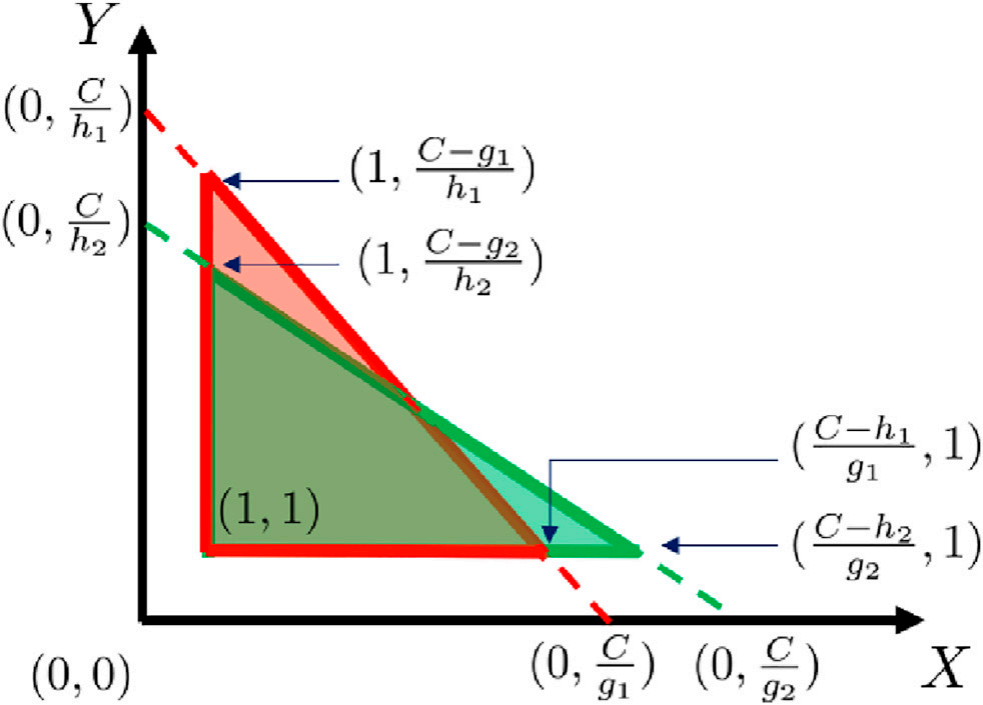
Illustration of computing $\text{PT}\left(n_{1}\right)$ and $\text{PT}\left(n_{2}\right)$. The red triangle is $S_{1}=\left\{\left(x,y\right)|h_{1}y+g_{1}x<C,x\in \left[1,H_{\max},y\in \left[1,H_{\max}\right]\right]\right\}$, and the green triangle is $S_{2}=\left\{\left(x,y\right)|h_{2}y+g_{2}x<C,x\in \left[\left[1,H_{\max}\right],y\in \left[1,H_{\max}\right]\right]\right\}$.

Terms in the above inequality can be computed by integration of the probability density function,

$$\underset{S_{1}}{\iint }\rho_{X,Y}\left(x,y\right)dxdy<\underset{S_{2}}{\iint }\rho_{X,Y}\left(x,y\right)dxdy,$$

where $S_{1}=\left\{\left(x,y\right)|h_{1}y+g_{1}x<C,x\in \left[1,H_{\text{max}}\right],y\in \left[1,H_{\text{max}}\right]\right\},S_{2}=\left\{\left(x,y\right)|h_{2}y+g_{2}x<C,x\in \left[1,H_{\text{max}}\right],y\in \left[1,H_{\text{max}}\right]\right\}$. By Assumption 5.2, $X\leq \frac{C}{g_{\text{min}}},Y\leq \frac{C}{h_{\text{min}}}$, and therefore $S_{1},S_{2}$ are two triangles, as shown in Figure 2. Due to the uniform distribution of X,Y, the above integration can be easily computed by multiplying area of $S_{1},S_{2}$ with the joint distribution $\rho_{X,Y}\left(x,y\right)$, which is a constant. Hence,

$$\frac{1}{2}\rho_{XY}\left(\frac{C-g_{1}}{h_{1}}-1\right)\left(\frac{C-h_{1}}{g_{1}}-1\right)<\frac{1}{2}\rho_{XY}\left(\frac{C-g_{2}}{h_{2}}-1\right)\left(\frac{C-h_{2}}{g_{2}}-1\right)$$

which implies $K\left(n_{1}\right)>K\left(n_{2}\right)$.

Let the ordered sequence Γ denote a path connecting the start node s with the goal node g in the graph G. Define $K_{\max}\left(\Gamma \right)$ as the highest key function value of all nodes on the path Γ, that is, $K_{\max}\left(\Gamma \right)=\underset{n\in \Gamma }{\max}K\left(n\right)$.

Lemma 5.7: The optimal path minimizes $K_{\max}\left(\Gamma \right)$ over all paths in the graph.

 PROOF: Let $\Gamma^{*}$ denote the optimal path that maximizes $\mathrm{Pr}\left(h^{*}\left(n\right)+g^{*}\left(n\right)<C\right)$. Suppose that there is a path Γ′ that is different from the optimal path $\Gamma^{*}$ with $K_{\max}\left(\Gamma \prime \right)<K_{\max}\left(\Gamma^{*}\right)$, then there exists n′∈Γ′, and $n\in \Gamma^{*}$ that satisfies K(n′)<K(n). By Lemma 5.6, PT(n′)>PT(n), indicating that $\mathrm{Pr}\left(h^{*}\left(n\prime \right)+g^{*}\left(n\prime \right)<C\right)>\mathrm{Pr}\left(h^{*}\left(n\right)+g^{*}\left(n\right)<C\right)$, which contradicts the assumption that $\Gamma^{*}$ is the optimal path. Hence, a path is the optimal one if it minimizes $K_{\max}\left(\Gamma \right)$.

Conversely, let $\Gamma =\underset{\Gamma \prime \prime \in G}{\arg\;\min\;}K_{\max}\left(\Gamma \prime \prime \right)$, then for all Γ′ that is different from Γ, for all n∈Γ,∃n′∈Γ′, such that K(n)<K(n′). Thus by Lemma 5.6, PT(n)>PT(n′) for n′ in any arbitrary path that is not Γ in the graph, and hence Γ that minimizes $K_{\max}\left(\Gamma \right)$ is the optimal path.

Theorem 5.8: When a feasible solution exists, the proposed algorithm terminates if and only if an optimal path is found.

 PROOF: Algorithm 3 can only terminate by finding the goal node, or after depleting the OPEN set. However, the OPEN set can never be empty before termination if there is a feasible path from s to goal point. Hence Algorithm 3 must terminate by finding a goal point.

Next we show that Algorithm 3 terminates only by finding an optimal path to the goal node. Suppose that the algorithm terminates by finding a path, Γ′ other than the optimal path $\Gamma^{*}$, then by Lemma 5.7, $K_{\max}\left(\Gamma^{*}\right)<K_{\max}\left(\Gamma \prime \right)$, that is, there exists n′∈Γ′, $n\in \Gamma^{*}$ such that K(n)<K(n′). Thus during the propagation process, Algorithm 3 would have selected n for expansion rather than n′, contradicting the assumption that the algorithm terminates by finding Γ′. Hence the algorithm must terminate by finding the optimal path to the goal node.

### 5.3 Complexity Analysis

In our analysis, we derive the worst case running time for Algorithm 3, and compare it with dynamic programming based planning methods, such as A*, to demonstrate the computational efficiency of the proposed planning algorithm. Let us suppose the flow field forecast is available on N×N grid points in the deployment domain, and suppose that the domain is partitioned into M cells by performing Algorithm 1.

To derive the worst case running time of the proposed algorithm, we first consider the partitioning. Since one junction must be formed by the boundary of at least two cells, the total number of junctions cannot exceeds M(M−1), and hence the total number of nodes in the graph is at most M(M−1). In one iteration process, the sorting operation (line 13), and computation of the key function, the minimum branch cost, and the heuristics (line 29, line 23 and line 27 are performed for one time. Suppose that the OPEN set is implemented using a heap data structure, the worst case running time of the operation in line 13 is O(log(M(M−1))). We assume that the computation of the key function, the minimum branch cost, and the heuristics can be performed in constant time. There can be at most M(M−1) iterations during the entire execution, before the OPEN set is depleted. Hence, the worst case running time of Algorithm 3 is O(M(M−1)log(M(M−1)).

The worst case running time of A* is $O\left(2N^{2}\log N\right)$ (Nilsson, 2014). Thus, the proposed algorithm is more computationally efficient than A* if $M\left(M-1\right)<N^{2}$, meaning that Algorithm 1 partitions the domain into less number of cells than the number of rectangular cells in the original gridded domain.

## 6 Experiment and Simulation Results

In this section, we provide the results of the implementation of our flow field modeling and path planning methods in a simulated experiment. First, we describe the simulated experimental set-up and recent field experiments, which serve as a strong test of the methods due to the magnitude and variability of the flow. We validate the proposed flow modeling algorithm by comparing the estimated flow model parameters generated from the proposed flow estimation algorithm with the glider estimated flow collected during the experiment. Based on the estimated flow model, simulation of the bounded cost path planning algorithm is performed, and its performance is compared with other AUV path planning algorithms.

### 6.1 Experimental and Simulation Setup

Our study is motivated by the use of underwater gliders off the coast near Cape Hatteras, North Carolina, US as part of a 16-months experiment (Processes driving Exchange At Cape Hatteras, PEACH) to study the processes that cause exchange between the coastal and deep ocean at Cape Hatteras, a highly dynamic region characterized by confluent western boundary currents and convergence in the adjacent shelf and slope waters. Underwater gliders, AUVs that change their buoyancy and center of mass to “fly” in a sawtooth-shaped pattern, were deployed on the shelf and shelf edge to capture variability in the position of the Hatteras Front, the boundary between cool, fresh water on the shelf of the Mid Atlantic Bight and the warmer, saltier water observed in the South Atlantic Bight.

While the energy efficiency of the glider’s propulsion mechanism permits endurance of weeks to months, the forward speed of the vehicles is fairly limited (0.25–0.30 m/s), which can create significant challenges for navigation in strong currents. Use of a thruster in a so-called “hybrid” glider configuration can increase forward speed to approximately 0.50 m/s, but at great energetic cost. The continental shelf near Cape Hatteras is strongly influenced by the presence of the Gulf Stream, which periodically intrudes onto the shelf, resulting in strong and spatially variable flow that can be nearly an order of magnitude greater than the forward speed of the vehicle (2+ m/s). With realistic estimates of the spatial and temporal variability of the flow, path planning can provide a significant advantage for successful sampling.

**FIGURE 3:**
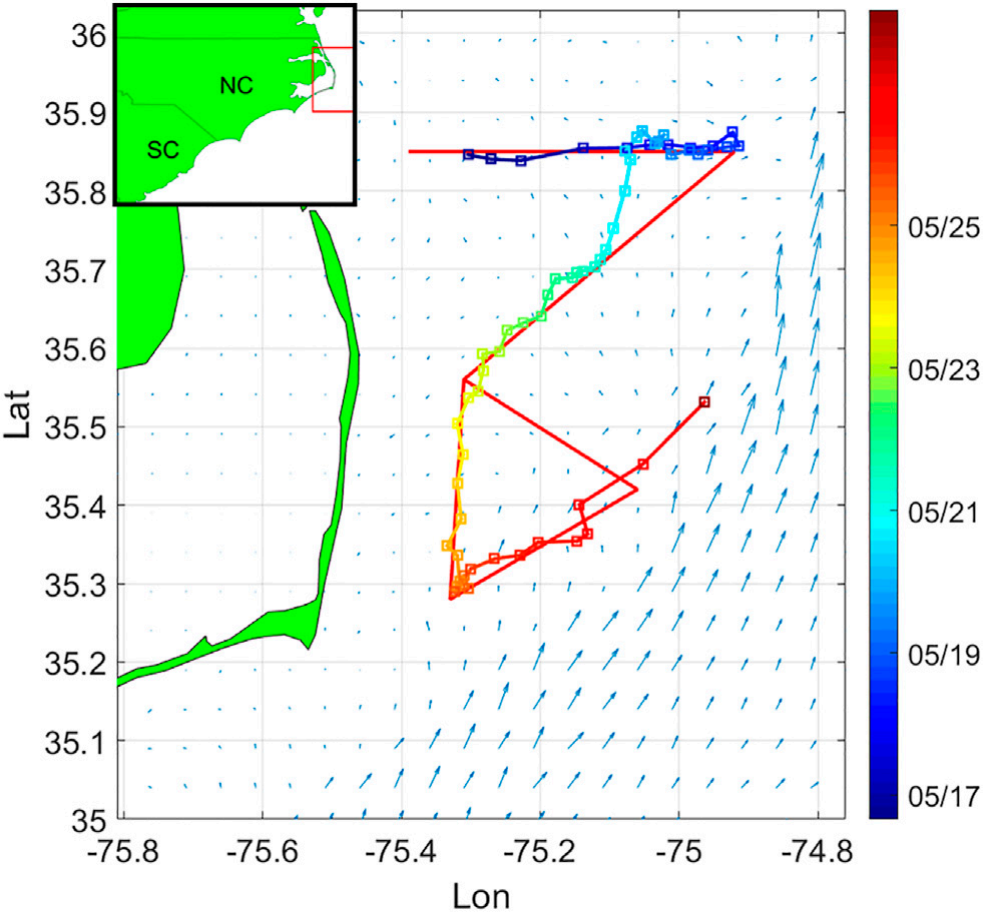
Survey domain near Cape Hatteras. The curve represents glider trajectory during the first PEACH deployment. The red line path is the pre-assigned sampling pattern. Squares denote the glider surfacing positions along trajectory, and color of the trajectory depicts timestamps. The arrows represent the NCOM-predicted flow field at the starting time of the deployment.

We deployed one glider off Oregon Inlet, NC May 16, 2017 and recovered it 14 days later. For its mission, the glider was initially tasked to sample along a path with offshore, inshore, and triangular segments designed to sample critical flow features (Figure 3), and was not used with a thruster. The glider surfaced approximately every 4 h to update its position with a GPS fix, communicate with shore, transmit a subset of data, and most importantly, receive mission updates and commands to adapt sampling.

### 6.2 Flow Modeling Using Glider Experimental Data

In this example, we present flow modeling results using the proposed flow partition and parameter estimation methods.

**FIGURE 4:**
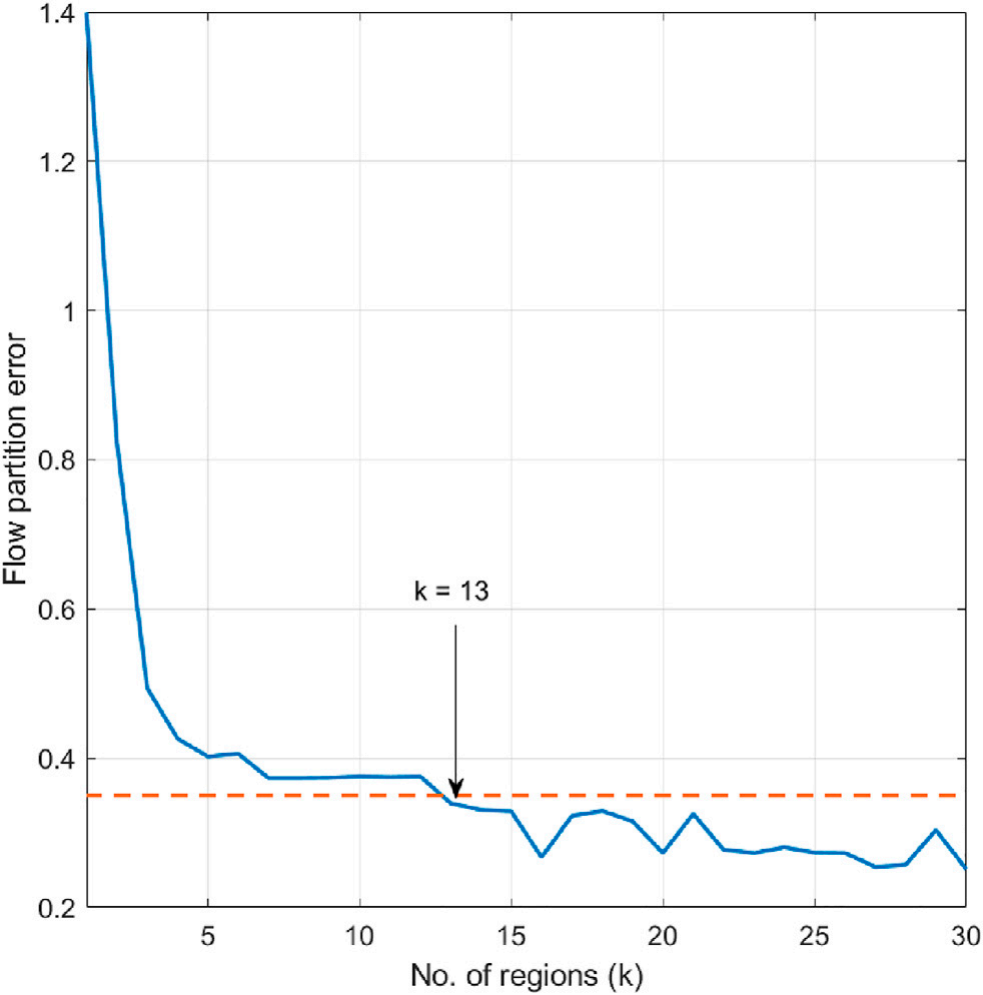
Flow partition error when the number of cells is set as k=1,…,30.

**FIGURE 5:**
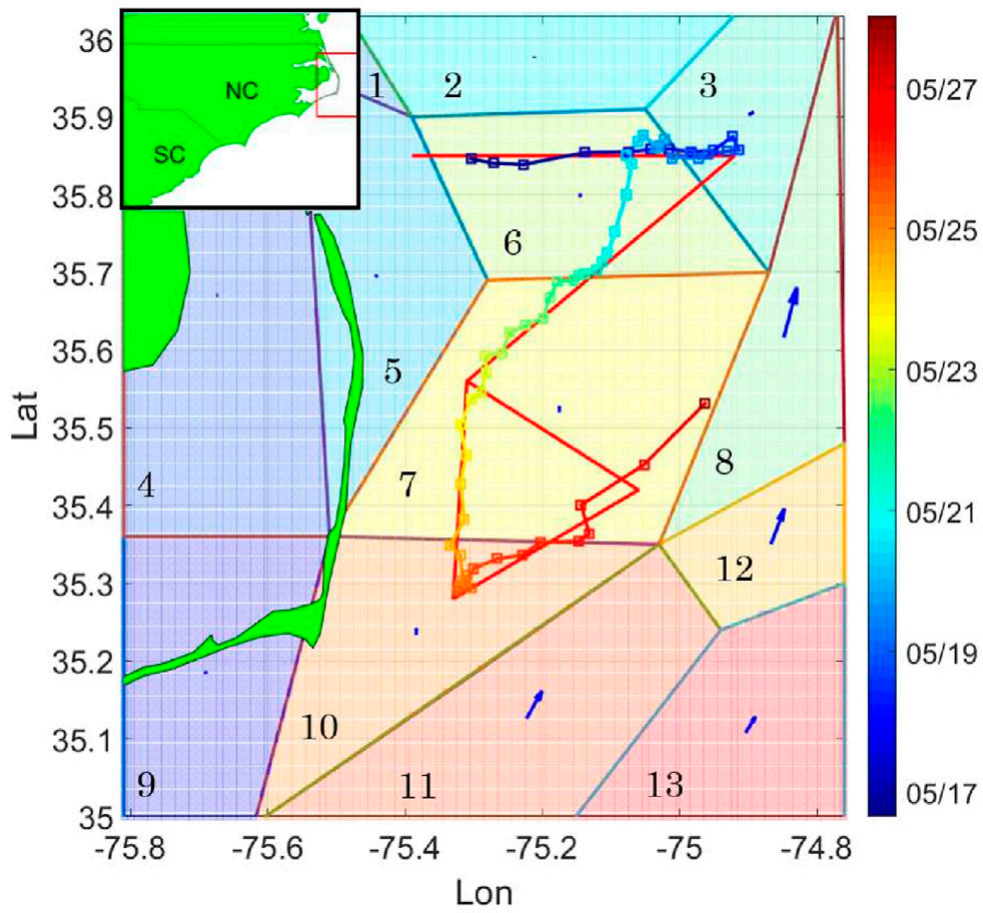
Partitioned cells of the survey domain. The polygons are the partitioned regions. The blue arrows represent uniform flow speed in each of the cells generated from the proposed algorithm.

The flow map forecast is given by a 1-km horizontal resolution version of the Navy Coastal Ocean Model (NCOM, Martin, 2000), made available by J. Book and J. Osborne (Naval Research Laboratory, Stennis Space Center, United States). In the domain of interest, the ocean model flow forecast is given at 106×106 rectangular grid points. Tidal flow accounts for much of the short-term (<24 hour) temporal variation of the flow field. Hence the partition time interval is taken over multiple periods of the largest tidal constituent, the lunar semidiurnal $M_{2}$ tide (period 12.42 h). Maximum flow speed in this area is 2.2788 m/s, approximately 7.5 times the vehicle speed, and 4.5 times the speed of a hybrid glider using a thruster. We set the upper bound for flow partition error to be 0.35 m/s, which is about 15% of the maximum flow speed in the domain. Figure 4 describes the flow partition error in the case of different selection of cell number. Since the flow partition error goes below the upper bound when k=13, the number of cells is chosen as 13. We smooth the cell boundaries into straight lines using the Least Mean Square method. Even though smoothing the cell boundaries might overlook more detailed spatial variability of the flow field, it helps to reduce the computational cost of solving the planning problem, specifically, in solving eq. 17 and eq. 20. The partitioned flow field is shown in Figure 5. Comparing Figures 3, 5, it can be seen that the proposed algorithm captures the major spatial variation of the flow field, by separating the high speed flow regions from the area where the flow is at lower speed.

**FIGURE 6:**
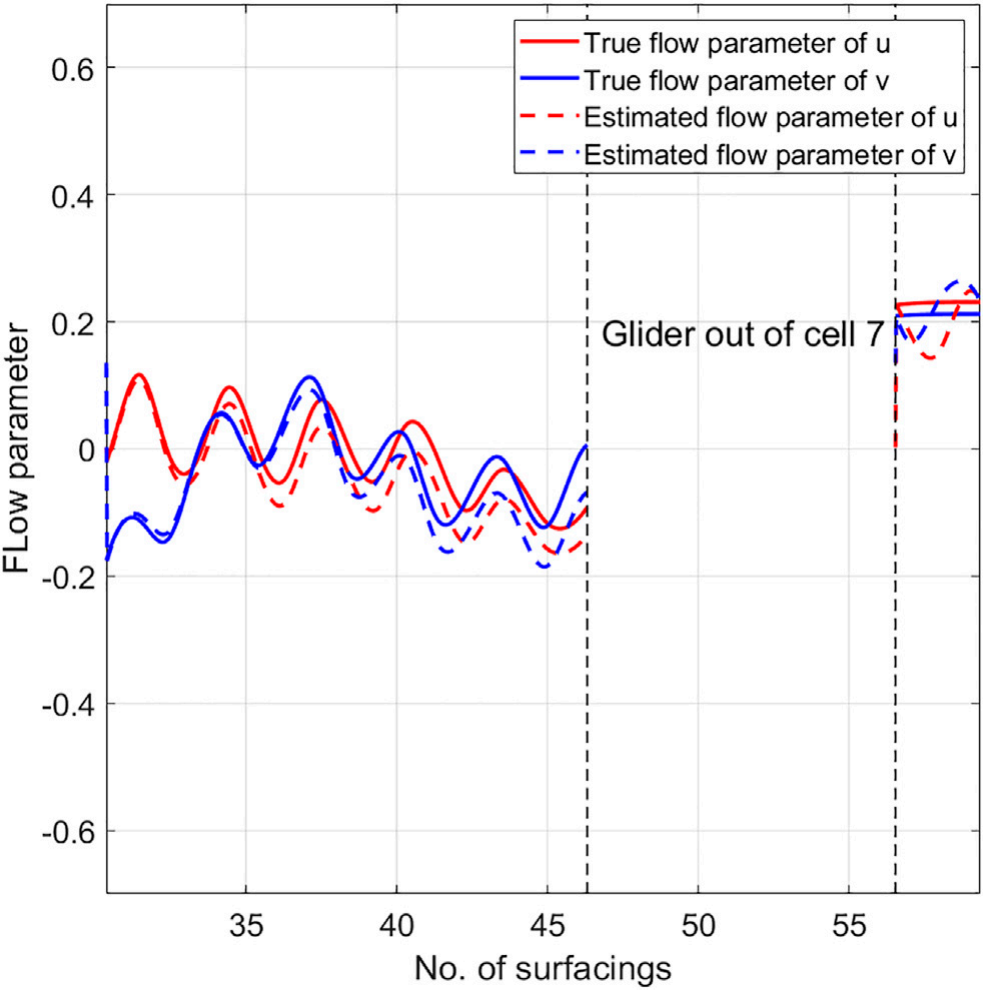
Estimated flow parameters and the ground truth value in cell 7.

At each surfacing, the vehicle position is given by the GPS location. When the glider is underwater, we use linear interpolation to estimate the heading and vehicle position. The vehicle’s forward speed is zero when it is at the surface of the ocean, and the vehicle drifts freely with the surface current. This violates the constant forward speed assumption stated in Assumption 2.1. Hence, we remove the segment of data when the vehicle is drifting at surface, and then compute the estimated flow parameters by the proposed algorithm. Glider speed is initialized to be 0.3 m/s, while the flow parameters are initialized by the flow vectors found by partitioning the NCOM data. Since the vehicle trajectory crosses cell 3, 6, 7, 10, and does not enter other cells, the glider trajectory does not satisfy persistent excitation condition described in Lemma 5.3. Hence only the flow parameters in cells 3, 6, 7, 10 can be updated by the adaptive updating law, while the flow parameters in the rest cells remain to be the initial value. To justify performance of the proposed flow parameter estimation algorithm, we use the ADCIRC (Advanced Circulation) model output (Luettich et al., 1992) to model the tidal flow component, and derive the non-tidal glider estimated flow speed by subtracting ADCIRC reported flow from the flow parameter estimate. The de-tided glider estimated flow speed is considered as the ground truth of flow parameters in the corresponding cells. The root mean square error (rmse) between the estimated parameter and the ground truth in cell 3, 6, 7, 10 is shown in Table 1. It can be seen that in all of the four cells, the estimated flow parameters is in good agreement with the true flow parameters. The rmse in all of the four cells is within 5% of the maximum flow speed in the domain. Figure 6 shows the comparison between the estimated flow parameters and the true flow parameter value in one of the cells that the glider trajectory visits. It is shown that in cell 7, the estimated flow parameter matches well with the true value.

**TABLE 1:** Root mean square error between the estimated and the true flow parameters.

Cell number	Flow parameter	Estimation error (m/s)
Cell 3	W-E flow	0.0558
	N-S flow	0.0082
Cell 6	W-E flow	0.0526
	N-S flow	0.0831
Cell 7	W-E flow	0.0415
	N-S flow	0.0388
Cell 10	W-E flow	0.0961
	N-S flow	0.0975

### 6.3 Bounded Cost Path Planning

In this example, we present simulation results of AUV bounded cost path planning. Since the flow field is of high speed in the domain of interest, we assume that the glider is sampling the domain using combined propulsion of buoyancy and thrusters for the operation. Hence the AUV through-water speed is set to be 0.5 m/s. The simulations are run on a core i7 at 1.80 GHz PC with 32GB RAM.

**FIGURE 7:**
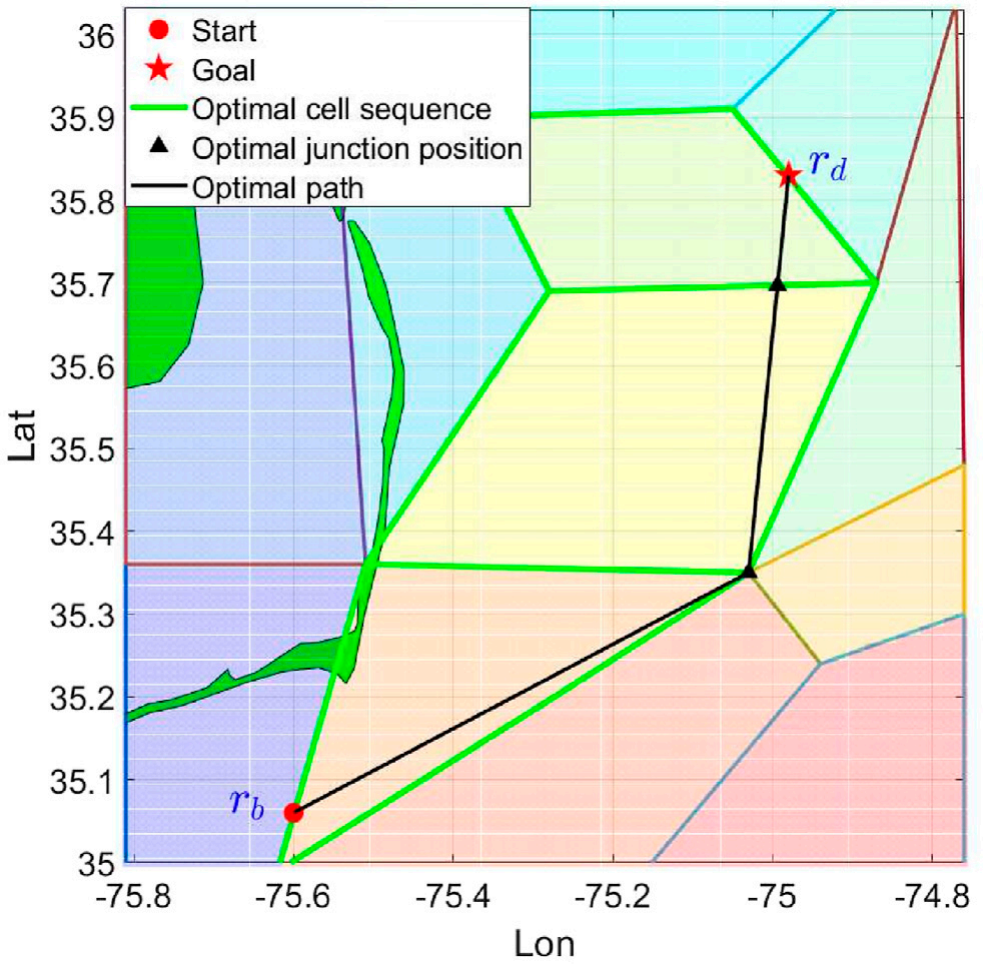
Example of simulation case. The resulting path is computed by the proposed method.

Figure 7 shows one example of the proposed bounded cost path planning method. The start position and goal position is assigned as (−75.60,35.06) and (−74.98,35.83) in longitude and latitude, respectively. Upper bound of the travel time is set as 72 h. The travel cost of the resulting path is 62.650 h, which satisfies the bounded cost constraint. As shown in the figure, the generated path makes a detour and takes advantage of the strong northward ocean flow to travel to the goal position.

We perform A* (Carroll et al., 1992) and Level Set Method (LSM) (Lolla et al., 2014) as comparison to the proposed method. 15 test cases are generated in total. Each test case T={Start,Goal,d} is built by first assign the distance between the start and the goal node d to be 20 km, 50 km, 80, or 100 km, then randomly place the Start point in the domain, and select the Goal node so that the distance to the start node is d. The computation time column in Table 2 shows comparison of the averaged computational time for A*, LSM, and the proposed algorithm. Table 3 presents the post-hoc analysis results of the simulation. The post-hoc analysis rejects null hypotheses of the same performance, i.e. the proposed algorithm spends less computation time to solve the planning problem than the A* and the LSM method, for all different scenarios of d. The difference between the three algorithms is due to the number of nodes in the graph. By partitioning the domain into 13 cells, the proposed algorithm searches for the optimal path in a graph with only 13×12 nodes, while both the A* and LSM algorithm searches for the optimal path in a domain containing 106×106 nodes. Thus, the computational cost of the proposed algorithm is significantly lower than the other two methods.

**TABLE 2:** Computation time comparison of A*, Level Set Method, and the proposed algorithm. Avg. comp. time represents the averaged computation time for each simulation scenario, and STD comp. time represents the standard deviation of the computation time. % of increase describes the percentage increase in the computation cost when d increases.

Method	d (km)	Avg. Comp. Time (s)	Std comp. Time	% Of increase
Algorithm 3	20	0.1576	0.0365	—
	50	0.1603	0.0385	1.7%
	80	0.2796	0.1127	77%
	100	0.4276	0.2318	171%
A*	20	1.6776	0.5199	—
	50	7.4376	0.7692	343%
	80	11.8376	1.3216	605%
	100	14.9040	1.1900	788%
LSM	20	86.8376	9.4397	—
	50	145.7043	30.0730	67%
	80	210.6376	23.4036	142%
	100	241.9043	25.6065	178%

**TABLE 3:** Post-hoc analysis of simulation comparison between the proposed method, A*, and LSM. The mean and STD of difference describe the mean and standard deviation of the computation time difference between the proposed method and the two other methods. The significance level is set as α=0.05 when computing the *p*-value.

Method	d (km)	Mean of difference	Std of difference	t-score	*p*-value
A*	20	1.5200	0.5108	11.53	$2e^{-8}$
	50	7.2773	0.7744	36.40	0
	80	11.5580	1.2980	34.49	0
	100	14.4767	1.1168	50.20	0
LSM	20	86.6800	9.4511	35.52	0
	50	145.5440	30.0805	18.74	0
	80	210.3580	23.4411	34.76	0
	100	241.4767	25.6747	36.43	0

Further, we compare the percentage of increase in the computation time when d increases. In Table 2, the % of increase column shows the increase in the computation cost when d increases from 20 to 50,80, and 100, respectively. The percentage is calculated by considering the computation time of each algorithm when d=20 km as the base time, and divide the increase of computation time when d scales up by the base time. It can be seen that when the domain of interest scales up, the computational cost of the proposed algorithm has the least increase, compare with A* and LSM. This is because as d increases, both A* and LSM have to expand significantly more nodes before finding the optimal solution. For the proposed algorithm, the number of nodes to be expanded stays relatively constant as d increases. Hence, its computation cost does not increase as much as A* and LSM as the domain scales up.

It is worth mentioning that the proposed algorithm achieves decreased computation cost by compromising the path quality. Even though optimality of the planned path is guaranteed in the partitioned flow field, as shown in Theorem 5.8, the planned path may not be optimal in the actual flow field, since the partitioned flow field is different from the actual flow field. We identify the compromised path quality as the major constraint of the proposed algorithm.

In cases where the domain is larger, Algorithm 1 may still result in large number of cells, leading to increased computation cost in solving the bounded cost planning problem. In such scenarios, stochastic optimization methods, such as the differential evolution method, may be helpful in further reducing the computation cost of solving the planning problem. We refer to a survey paper on the differential evolution methods (Das et al., 2016) for this matter.

## 7 Conclusion

In this paper, a bounded cost path planning method is developed for underwater vehicles assisted by a data driven flow field modeling method. The main advantage of the proposed modified PTS method is that it is more computational efficient comparing to A* and LSM in solving AUV planning problem in time-invariant 2D fields, as demonstrated by the simulation result. Major limitation of the proposed algorithm is the compromised solution quality, resulting from the model reduction error introduced by the flow partition process. The proposed method has the potential to be extended to other path planning applications where the task performance is sensitive to planner’s computational efficiency. Future work will include performing the proposed method in real glider deployments, to compare the planned trajectory with the real mission trajectory, where drift and time-varying fields happen. Future work will also include comparing the proposed method with other algorithms, such as the differential evolution algorithms.

## Data Availability

Publicly available datasets were analyzed in this study. This data can be found here: https://www.ncdc.noaa.gov/data-access/model-data/model-datasets/navoceano-ncom-reg. The flow map data analyzed in this work is given by the Navy Coastal Ocean Model made available by J. Book and J. Osborne of Naval Research Laboratory, Stennis Space Center, US.
